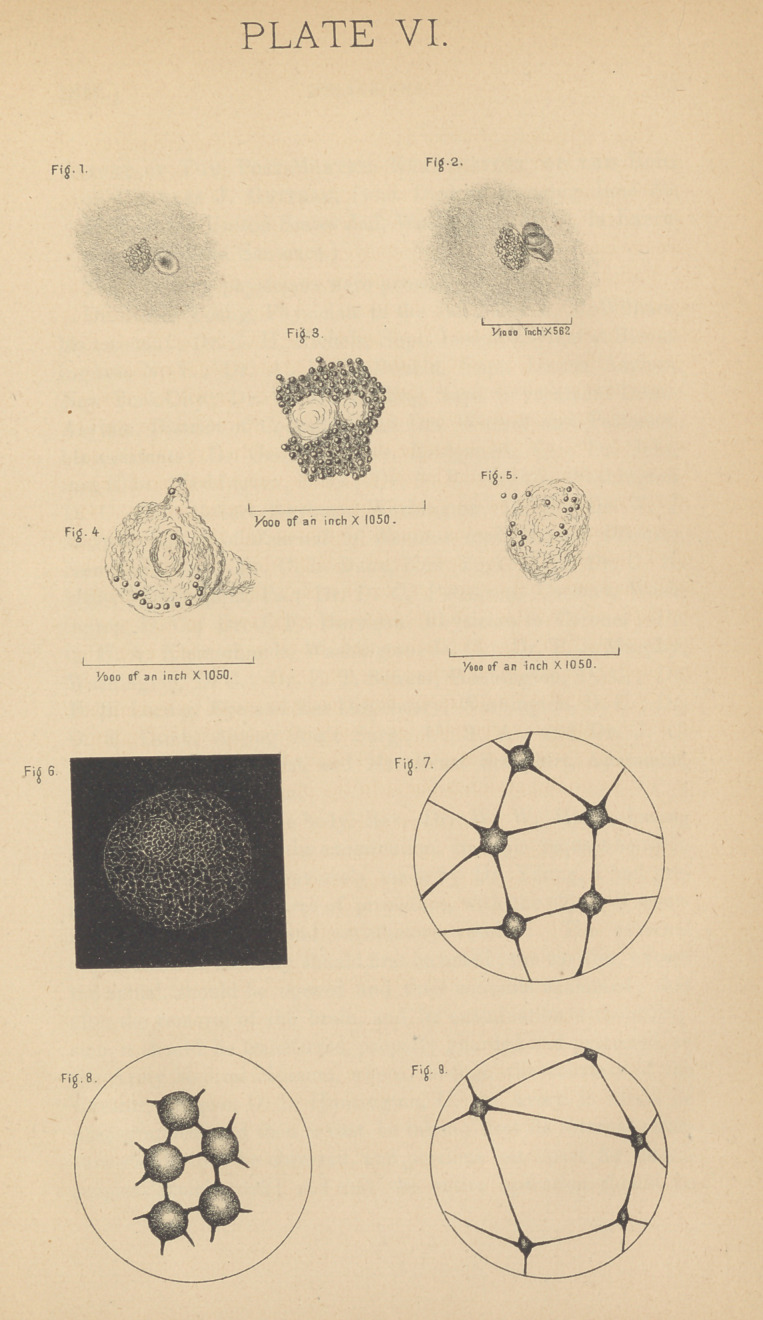# A Study of Blood

**Published:** 1882-07

**Authors:** Lester Curtis

**Affiliations:** Chicago, Ill.


### Selections.

A STUDY OF BLOOD. By LESTER CURTIS, M.D., Chicago, Ill.

In the transactions of the American Medical Association for the year 1875 appeared a paper by Dr. Louis Elsberg, of New York City, entitled, "Notice of the Bioplasson Doctrine." It gave the results of some researches carried on in the laboratory of Dr. Carl Heitzmann, formerly of Vienna, but now of New York, and was concerned with the structure of the cell.

According to these observations, every cell contained fine fibers which branched and inosculated and formed a close net-work, which filled every part of the cell. This network was supposed to exist in all cells, its contractions and relaxations to constitute the amœboid movements of cells; in short, it was supposed to be the living matter of the cell. Before this time Dr. Heitzmann himself had described the same thing in a paper before the Vienna Academy of Sciences, in the year 1873, entitled "*Bau des Protoplasms.*"

Very little notice was taken of the subject at first, at least in this country. But of late several papers on the subject in Europe, and especially its favorable notice in Klein's Atlas of Histology, have given it more prominence.

The subject is one which differs from ordinary histological subjects, such as the structure of a gland, or the termination of a nerve, which persons interested in some other department of biology can afford to ignore. It is concerned with the very foundations of physiology and histology. It is of as much interest to the botanist, the diatomist, or the student of life in any form, as to the student of human physiology and histology. If the doctrine prove true, we shall have made a long step in advance in the understanding of cell growth, and shall have to unlearn many things which, until now, we had supposed settled. As I am occupying a position where I am expected to have some knowledge of my own about such things, I have looked into the matter for myself.

In order to make sure of pursuing the right course, I wrote to Dr. Heitzmann, asking him his method of demonstrating the structure, and received the following reply:*

NEW YORK, June 30, 1879.

LESTER CURTIS, M.D.,—*Dear Doctor:* In reply to your favor, dated June 27, 1879, I have to say as follows: The lens for seeing the structure of protoplasm must be a first-class 1-10 Immers., such as I use of Verick's, Hartnack's, Grunow's and Tolles' manufacture. Just the reticular structure itself is the best test for a lens, so far as my own experience goes.

Take a drop of pus, fresh, without adding anything, and you will see the wonderful structure in each pus corpuscle with great ease.

Prick your skin on the palmar surface of the thumb, transport the drop on a slide, and cover right away with a thin covering glass, the edges of which have been oiled, so as to prevent evaporation of the fluid. In the perfectly fresh blood you will see the structure in each colorless blood corpuscle.

Add to a drop of fresh blood a small drop of 40 per cent. solut. of bichromate of potash; this will, within 1 hour, extract the hæmoglobin, and you must succeed in seeing the reticular structure in each red blood corpuscle.

Keep ordinary yeast (Torula cerivisiæ) for a fortnight in a 30 per cent. solution of bichromate of potash, and examine a drop with a good 1-10 Immers. lens. You cannot fail in seeing the network in each oidium.

Take any protoplasmic body, best epithelium, cartilage, etc., best kept for a few weeks in ½ per cent. solution of chromic acid; take any fresh living protoplasm such as, for instance, an amœba from an infusion, and you must see what is to be seen with accuracy. If you fail, come to New York to my laboratory, top floor of my residence, and after two hours you may leave for Chicago, with the satisfaction that the net-work in the protoplasm is plain. About four hundred gentlemen have seen it so far in my place.

Yours truly, Dr. C. HEITZMANN,
No. 37 West Forty-fifth Street.

On receipt of this, I repeated the experiments as directed. The glasses I have used are a 1-10 immersion, made by Wales in 1874; a pretty good glass for one made at that time. It has, in my hands with sunlight and a slip of blue glass, gone through Möller's balsam *probe platte* without difficulty. I also used a

*I publish a careful copy of this and the following letter, by Dr. Heitzmann's permission.—L. C.

Powell & Lealand 1-16 made later in the same year. With this glass I have glimpsed the lines on the dry *amphipleura* by simple lamp-light, without any sub-stage appliances whatever. In Mr. Tolles' hands, by the use of his traverse lens, it showed the lines on number 20 of the *probe platte* fully as well as one of his earlier duplex 1-10s, although, since that time, I have seen the diatom better with one of his later 1-10s. I use a stand made by Bulloch, fitted with a Powell & Lealand achromatic condenser. I find that for making out delicate anatomical structures, with the use of high powers, the condenser is indispensable.

I began my investigation by the study of blood as directed by Dr. Heitzmann. I split off a thin film of mica and oiled the edges. I then pricked my finger with a needle, put a drop of blood on a slide and covered it immediately with the mica. I began the examination with the 1-10. On bringing a white corpuscle into view, I thought, at first, that I saw the net-work; a number of fine lines appeared, crossing the corpuscle in all directions, but the outlines of the red corpuscles in the field were indistinct. The glass had been used in examining objects protected with a thicker cover, and was not adjusted for such a thin film as I was then using. In order to improve the definition I turned the screw collar. As I turned, the outline of the corpuscle grew sharper, but the net-work became more indistinct. Finally, when the outline of the corpuscle came out clear, I could see nothing of the network. Instead, the corpuscle appeared to be covered with small nodules of unequal size, placed at irregular intervals.

I went through nearly the same experience when, in place of the 1-10, I put on the 1-16. At first, before the cover adjustment was right, an indistinct appearance resembling a network was seen. But, when the adjustment was such that the outline of the red corpuscles in the field was sharpest, the same thing was seen as with the 1-10, only with greater clearness. I could, with the 1-16, by toying with the fine adjustment, focus the top of the nodules, while the remainder of the corpuscle appeared in shadow; and then, by gently lowering the glass, I could bring the valleys into view and leave the tops of the nodules indistinct. By changing the direction of the light, I could make the nodules cast shadows in first one direction and then in another. With all the care I could use, I could get no other appearance of a network than I have described as showing when the adjustment of the glass was wrong.

Having an abscess handy in my own person, I next examined some fresh pus, first with the 1-10 and then with the 1-16, as I had done with the blood.

The pus showed very nearly the same as the white blood corpuscles, possibly more distinctly. Several of the pus corpuscles were packed full of dancing granules. The movement of these granules seemed to be independent of each other, and reminded me of small animalcules imprisoned in a narrow space. By fixing my attention on one of them, and watching it for some time, I have seen it change its location and travel nearly half way across the corpuscle before escaping from view.

I next put a drop of blood on a slide, and added to it a small drop of a 40 per cent. solution of bichromate of potash. After an hour or two, I examined the red corpuscles with the 1-10 and 1-16 as I had done with the fresh blood. Many of the corpuscles had lost a good part of their color. With the color, they had also lost the smooth surface which they usually possess in the fresh state. The edges were finely crenated, the surfaces were thrown into nodules and ridges, and all the corpuscles were pinched and shrivelled, and were much smaller than the fresh corpuscles, appearing as though acted upon by an astringent.

I took the trouble to measure seven of these corpuscles in a given area of one field of the microscope. They were as follows, measured in both directions, and given in parts of an inch :

| | | |
|---|---|---|
| (1) | 1–6,000 | 1–4,500 |
| (2) | 1–5,000 | 1–5,200 |
| (3) | 1–4,500 | 1–3,700 |
| (4) | 1–4,500 | 1–3,871 |
| (5) | 1–4,275 | 1–3,946 |
| (6) | 1–6,000 | 1–3,461 |
| (7) | 1–3,461 | 1–4,687 |

Here, also, I failed to get satisfactory evidence of the existence of a network; though the shadows between the ridges and elevations might resemble one.

I carried my investigations to this point, and, after carefully reviewing my work, to be sure I had made no mistake, I wrote to Dr. Heitzmann again. I told him how I had proceeded and what I had seen; I also enclosed some drawings of white corpuscles as they appeared to me, and some of red corpuscles, which had been acted upon by the 40 per cent. solution of bichromate of potash, and asked his interpretation. He sent me the following letter:

NEW YORK, Feb. 8, 1880.

DR. LESTER CURTIS. *My Dear Doctor*:—I have read your letter with great interest. It merely proves how difficult it is to learn microscopy as an autodidact without the assistance of a reliable teacher. The network is seen plainly when the peripheral contour is not plain; in a globular body the central portion shows the structure best when the periphery is out of focus. You evidently did not learn to discriminate between different layers in one and the same corpuscle, as illustrated by your sketches. You draw everything—in and out of focus. You should draw only what is clear and sharp in *one* focus. Look at the Histology Atlas by E. Klein and Noble Smith, first volume (1879. Lippincott publishers). E. Klein is the best microscopist of London, England; he draws the network even nicer than it really appears, and gives me credit for the discovery.

Purchase for a few cents the recently issued researches on red blood corpuscles, by L. Elsberg (1879. Putnam Sons, N. Y., publishers) and you will see and learn everything you desire. You evidently are a good, faithful man. Could not you come to New York in my laboratory? Here you would learn more in one day than you possibly can learn at home in months.

Yours truly,

DR. C. HEITZMANN.

On the receipt of this letter I was, of course, dismayed at my great presumption in attempting to make out the structure of a blood corpuscle with a 1-16th of an inch object glass; a task so much more difficult than resolving the *amphipleura* that no ordinary mortal may ever hope to accomplish it. But I could not resist the temptation to return to the subject. Accordingly, I re-studied the white corpuscle. I found that when I had, by careful manipulation, succeeded in focusing the bottom of the valleys, I could go no further, the slightest touch of the fine adjustment, the slightest pressure upon the limb of the instrument, even, would cause a blurring of the image. I tried again and again, and am obliged to confess that I cannot focus different planes of a white blood corpuscle.

While I was doing this my mind would keep reverting to the war once waged over the structure of diatoms; how the nodular surface of pleurosima angulatum was supposed to be covered with hexagons so strikingly similar, in some respects, to this network; and I remembered how the whole thing was cleared up when object-glasses and modes of examination became more perfect. The idea would keep coming to me that this network, also, was an optical illusion, explained in the same way.

I began a series of persecutions of my friends. I importuned almost every one that I knew, who had a microscope, to look over the subject and tell me what he saw. Some of these were persons of recognized skill in microscopic manipulation. Some, even, have a national reputation. Many were kind enough to do as I requested, but no one was able to see the network. Indeed, I have never seen any one who claims to have seen it. A few have allowed me to use their names. Dr. H. A. Johnson, of this city, carefully went over the ground with me, using a superb new Tolles' 1-10, and a new Zeiss 1-12 homogeneous immersion. He considers what I have described to be true appearances. Dr. Sternberg, of the United States Army, has studied the white blood corpuscle especially, with the best appliances at the command of the Government. He tells me that he has never seen such a network.

A professor in one of the medical colleges in this city, in one of his lectures, described the network, as given by Klein, as a new discovery. Later in his course he corrected this statement, and said that subsequent investigation had convinced him that the network was an optical illusion. This was done independently of my work.

I might increase the list, but do not feel justified in using the names without permission.

As to Dr. Heitzmann's criticism of my drawing, I will only say that I had supposed a drawing for scientific purposes to be good, not as it left out objectionable details and improved upon those represented, but just in proportion to the accuracy with which it reproduced the appearances seen. It has always been my aim to represent objects as nearly like what I saw as I could. Any other course has seemed to me to be not only bad drawing, but absolute dishonesty.

While engaged in these studies I met with certain bodies which I have not seen described elsewhere just as I have seen them, although Dr. Osler and others have spoken of having seen something like them in the blood of the lower animals.

The bodies are minute granules. They can be seen in blood, with a good high-power glass, without the addition of any reagent, but are best seen after staining. If, while studying blood with the microscope inclined, a drop of carmine staining-fluid be placed at the upper edge of the covering-glass, there will be seen bright red points sliding across the field of the microscope long before any other evidence of staining appears. As the staining-fluid comes down further, the points increase in number, until finally, when the field of the microscope has been entirely traversed, they are countless. Under a magnifying power of twelve hundred diameters, they appear as minute, round bodies. Their small size renders a measurement of them difficult; but I have estimated their diameter at from 1-20,000 to 1-40,000 of an inch. They stain deeply with carmine, and are highly refractile, the stained ones appearing as bright red points surrounded by a black rim. The width of this rim varies as the focus is changed; when they are slightly beyond the focus, the red point disappears, and they show as black dots.

I have seen these bodies in the blood of every person which I have examined, with one exception. I studied the blood of Mr. Griscom during a long fast which he underwent in Chicago. For part of this fast these bodies were absent. During their absence I observed many granular white corpuscles of exceptionally large size. They seemed to be composed of spherules of about the same size as the granules before described. The corpuscles often had an active amœboid movement. At this time the granules flowed out into the protruding portion in a kind of stream. Aside from this motion the granules were usually still. Occasionally, however, they had an independent motion, like the motions of the granules in the pus corpuscle before described. I have watched one of the granules move for a considerable distance through the corpuscle; the other granules at this time were vibrating uneasily like individual bees in a swarm. The corpuscle itself, with the exception of an almost imperceptible vibrating movement, remained still.

These corpuscles usually had one or more places destitute of granules, which resembled nuclei. The spots did not stain with carmine, and were unmistakable depressions. They seemed to me, therefore, to be places free from granules, rather than nuclei. Their floor showed a slightly uneven surface.

I studied these bodies for about two weeks, the time of the absence of the granules from the blood. After a time the granules began to return. At first they were of a low, refractive index, and stained faintly; gradually they assumed their usual appearance.

About the time of the reappearance of the granules, I noticed a change in the granular corpuscles. They stained more readily and more deeply with carmine than ever before; the places destitute of granules became larger and more numerous, and I began to see appearances as though granules were leaving them. On two occasions I saw bodies which presented all the appearances of the other granular corpuscles except that the nuclear-like space had enlarged to such an extent that the body was nearly destitute of granules. Many granules were seen in the act of passing out from the corpuscles, and many were seen near the corpuscles, as though they had just left them.

The conclusion from these facts seems to me to be warranted, that the granular corpuscles, at least, are composed of minute round bodies held in a stroma, and that, possibly, they may be the source of the granules found in the blood.

EXPLANATION OF PLATE.

Fig. 1. An ordinary white blood corpuscle, with one red corpuscle.

Fig. 2. An ordinary white blood corpuscle from another person, and two red corpuscles, lying one over the other.

Fig. 3. Granular white corpuscle from Mr. Griscom; granules beginning to leave the body.

Figs. 4 and 5. Granular corpuscles from Mr. Griscom, discharging their granules.

Fig 6. A white blood corpuscle referred to by Dr. Heitzmann; from Klein's Atlas of Histology.

Figs. 7, 8, and 9. Representations of the network from "The Structure and Other Characteristics of Colored Blood Corpuscles," by Louis Elsberg, page 46, referred to by Dr. Heitzmann.

Fig. 7. Represents the network at rest. Fig. 8. In extreme contraction. Fig. 9. In extreme extension. These figures cannot be magnified less than 20,000 diameters, and must, therefore, be diagramatic.

## Figures and Tables

**PLATE VI. f1:**